# Influence of zinc foliar spray on growth, some important physiological processes, yield and yield attributes of bread wheat under water stress

**DOI:** 10.1038/s41598-025-94728-1

**Published:** 2025-04-29

**Authors:** Hanan E. Ghanem, Doaa A. Hamza, A. A. Zain El-Abdeen, Walaa S. Elbatrawy, Helal M. El-Habashy

**Affiliations:** 1 Wheat Research Department, Field Crops Research Institute, Agriculture Research Centre (ARC), Giza, Egypt; 2 Seed Technology Research Department, Field Crops Research Institute, Agricultural Research Centre (ARC), Giza, Egypt; 3 Soil and Water Science Department, Faculty of Technology and Development, Zagazig University, Zagazig, Egypt

**Keywords:** Bread wheat, Membrane characteristics, Water content, Yield parameters, Physiology, Plant sciences

## Abstract

Wheat constitutes one of the foremost important food crops in nations worldwide today. However, drought is one of the main global climate change risks that reduces agricultural productivity and causes significant economic losses. Zinc is an important nutritional microelement and is crucial for crops to be drought-tolerant. A field experiment was conducted at the Experimental Farm of Ismailia Agricultural Research Station, Egypt over two consecutive seasons under sprinkler irrigation to investigate the impact of various zinc levels (0, 100, 250, and 500 ppm) on Misr 3 wheat cultivar subjected to normal (100% field water capacity) and water stress (50% field water capacity) conditions. A split-plot design was used with three replicates, with irrigation treatments and zinc treatments allocated in separate main and sub-plots. Water stress was found to have a negative impact on shoot and flag leaf biomass, chlorophyll and carotenoids content, water relations and membrane characteristics. Also, drought significantly reduced yield and yield components. On the other hand, a gradual increase of Zinc up to a high level of 500 ppm alleviated water stress on wheat plants, thereby increasing the values of shoot fresh mass, shoot dry mass, flag leaf fresh mass, flag leaf dry mass, total chlorophyll, carotenoids, RWC, MSI and nutrient uptake. Moreover, all Zinc levels, especially 500 ppm, increased grain yield to 66.95–65.3%, yield attributes and the grain’s chemical composition (the relative content of wheat grain total protein, wet gluten, dry gluten and N P K Zn) under control and drought treatments. Therefore, foliar Zinc application, particularly at 500 ppm, can improve wheat plant growth, yield and grain nutrients beneath control and/or water stress conditions.

## Introduction:

Wheat (*Triticum aestivum* L.), represents one of the world’s greatest cereal crops, and global consumption is increasing^1^ as a consequence of population growth^2^, nutritional value, and interchangeability among different cereals^3^. Furthermore, Wheat is an excellent source of proteins, carbohydrates, dietary fiber and numerous bioactive substances that benefit people’s health^4^.

Drought as an abiotic stress severely impairs the morphophysiological properties^5^, plant-water relationships^6^, as well as yield criteria^7^ of stressed wheat plants. Cell membrane leakage or stability is a physiological measure that is frequently used to assess a plant’s tolerance to water stress^8^. As a result, protecting cell components function and structure during water stress is critical to maximizing plant yield in the face of changing climate^9^. Drought-induced reductions in grain production and nutritional values result in an additional burden, resulting in nutritional and food poverty in humans as well^10^.

In this regard, micronutrient fertilizers can improve plant water tolerance by enhancing plant defense mechanisms. Zinc has been recognized as a vital trace mineral that plays an important physiological effect on biological processes such as enzyme activation in many biochemical processes, protein synthesis and function, leaf photosynthesis, tolerance to stress, plant metabolism control, and plant development and production^11^. Zn has been shown to improve water potential, preserve cell integrity, and detoxify free radicals caused by drought in plants^12^. Apart from its function in the synthesis of chlorophyll and carotenoids, zinc has a substantial impact on metabolism and is linked as a co-factor to other enzymes. Zinc applied topically has been shown to boost crop yields^13^.

Reduced Zn levels in plants have been linked to drought stress brought on by lower soil water levels, which in turn limits root growth^14^. Zn treatment during a drought would affect crop quality and output. It has a vital function in regulating stomata and ionic balance in crops to reduce the harmful effects of drought^15^. Worldwide, it is estimated that 50% of grain crops are cultivated on soils low in Zn^16^.

Zinc fertilizer is frequently used to increase crop production, Zn concentration, and edible component quality^17^. Zinc is recognized to have a significant impact on fundamental plant functions as protein quality, nitrogen metabolism, enzyme activation, and chlorophyll synthesis^18^. Zinc has been shown to increase wheat yield and yield components^2^, and when applied properly, zinc has been demonstrated to increase wheat plants’ water use efficiency^19^.

Zinc concentration in wheat is typically low, and it is more deficient in newly sand soils. Applying foliar zinc sprays under water stress can improve crop resilience to water stress and raise zinc concentrations to ideal levels. With the aforementioned considerations in mind, the purpose of this field experiment was to demonstrate the active role that zinc plays in fostering tolerance by stimulating growth, some physiological functions, nutrient uptake, increasing the productivity of bread wheat under water stress.

## Materials and methods

### Plant growth conditions

A field trail was conducted at The Experimental Farm of Ismailia Agricultural Research Station (Lat. 30° 35′ 30″ N, Long. 32° 14′ 50″ E, 10 m above the sea level), Egypt over two consecutive seasons under sprinkler irrigation using Misr 3 wheat cultivar. The pedigree and source of the wheat grains were obtained from the Wheat Research Department, Field Crops Research Institute, Agricultural Research Center, Egypt (Table 1).

**Table 1:** Pedigree, origin, and selection history of the studied wheat cultivars.

Cultivar	Origin	Released year	Pedigree
Misr 3	Egypt	2010	Oasis/SKauz//4* Bcn/3/2*pastor

A split-plot design in RCBD was used with three replicates. Irrigation and zinc treatments were allocated in separate main and sub-plots. Irrigation treatments were done at two intervals: 50% of field capacity for water stress and 100% of field capacity for control, immediately after the accomplishment of germination. The field capacity was estimated according to Sarkar^20^. Before sowing at a depth of 30 cm for which the bare soil area was filled with extra water and covered with a plastic cover. Two days later, soil samples were taken from this depth, and the moist soil was weighed and dried in an oven set to 105 °C until it reached a constant weight (about 24 h later). The dry soil was then weighed to determine the soil moisture at 100% field capacity. In the case of a water stress, irrigation was restricted to 50% field capacity.

#### Operating time of irrigation system

Operating time for the solid-set sprinkler irrigation system was calculated using the following equation

$${\text{Operating}}\;{\text{time}} = \frac{{{\text{Bulk density }}\left( {{\text{g}}\;{\text{cm}}^{{ - 3}} } \right) \times {\text{soil}}\;{\text{moisture}}\;{\text{at}}\;{\text{field}}\;{\text{capacity }}\left( \% \right) \times {\text{soil}}\;{\text{depth}}\left( {\text{m}} \right) \times {\text{Area}}\left( {{\text{m}}^{2} } \right)}}{{{\text{Sprinkler flow rate}}\left( {{\text{m}}^{3} \;{\text{hr}}^{{ - 1}} } \right) \times {\text{reciprocal}}\;{\text{of}}\;{\text{irrigation}}\;{\text{efficiency }}\left( \% \right)}}$$

$${\text{Operating}}\;{\text{time}} = \frac{1.65 \times 0.1 \times 0.3 \times 78.5}{{1.13 \times 1.25}} = 2\;{\text{hours}}\;45\;{\text{minutes}}$$

#### Irrigation treatments

The Irrigation treatments were divided into two levels; 50% and 100% of the field capacity as follows:

Control: a full irrigation which had 100% of the Field capacity with operating time of 2 h and 45 min.

Water stress: 50% of Field capacity with operating time of 1 h 23 min.

Four levels of zinc: 0 (control), 100, 250 and 500 ppm were foliarly applied using chelating Zn EDTA. Zinc Doses were chosen in light of reviews and prior studies. Foliar Application of Zinc was done according to^21^. The foliar spraying was applied at 10 pm in two equivalent dosages: the first was added at the vegetative growth stage and the second one was added at seven days before the heading stage. At seed bed preparation, thirty kg of P_2_O_5_/fed were used, twenty four kg of K_2_O/fed were applied to plants during the tillering stage in two equal doses within ten days intervals and 120 of kg N fed^−1^ were added in five equal doses.

### Soil assessment

Before planting the wheat crop, the soil was sampled with an auger to record its chemical and physical characteristics. Soil specimens were placed in bags of polyethylene, tagged, and transported to the Ismailia Agricultural Research Station’s soil and water testing laboratory for assessment. Details of various physiochemical properties were included in Table 2. Climatic conditions, such as air temperature (°C) and relative humidity% (RH) were monitored on a daily basis and their every month mean values were determined (Table 3).

**Table 2:** Soil physical and chemical characteristics just before both wheat crop planting seasons.

Parameters	Season 1	Season 2
Mechanical analysis		
Sand (%)	89.20	88.80
Clay (%)	3.78	3.98
Silt (%)	7.02	7.22
Texture grade	Sandy	Sandy
Chemical analysis		
pH (1:25)	7.89	7.90
EC (dSm^−1^)	0.4	0.41
Organic Matter (%)	0.47	0.49
Organic C (%)	0.27	0.29
CaCO3	0.32	0.33
Soluble anion meq/100g soil		
CO_3_^2−^	–	–
HCO_3_^−^	0.5	0.53
Cl^−^	1.92	1.93
SO_4_^2−^	1.45	1.45
Soluble cation meq/100g soil		
Ca^2+^	1.22	1.23
Mg^2+^	0.63	0.65
Na^+^	1.84	1.84
K^+^	0.18	0.19
Available N P K Zn (ppm)		
N	20	20.2
P	4.5	4.6
K	57.18	57.21
Zn	0.08	0.09

**Table 3:** Climatic conditions of the experimental site during both wheat growing seasons.

Season Parameter Months	1			2			1	2	1	2
	Air temperature (°C)						RH		Rainfall (mm)	
	T_min_	T_max_	T_aver_	T_min_	T_max_	T_aver_				
November	16.37	23.57	19.79	17.57	26.67	22.12	61.5	62.9	1.4	1.7
December	11.36	22.44	17.22	10.41	19.24	14.825	59.7	64.9	6.3	5.6
January	9.82	21.2	16.7	6.82	15.7	11.26	63.3	67.3	0.1	8.1
February	11.67	22.06	16.33	9.87	19.96	14.915	61.97	64.07	22.4	8.4
March	11.1	23.7	18.2	9.6	21.69	15.645	59.9	53.7	0.8	5.6
April	13.09	29.6	21.86	14.49	31.4	22.945	44.9	40.6	0	0
May	17.10	32.2	24.65	15.9	28.4	22.15	39.9	44.9	0.5	0.3

T_min_: Minimum Temperature, T_max_: Maximum temperature, T_aver_: average temperature, RH: Relative Humidity.

At heading, a total of 10 samples were gathered from every treatment to evaluate shoot and flag leaf biomass. Furthermore, just three samples from each treatment were collected for flag leaf biochemical analysis at heading stage after two weeks of foliar application of zinc. For both of the seasons, data was collected and mean values were estimated.

### Growth parameters

- Shoot fresh weight (g)
- Shoot dry weight (g)
- Flag fresh weight (g)
- Flag leaf dry weight (g)

### Membrane stability index (MSI)

Sairam et al.^22^ method with a few modifications was used for gauging MSI. Leaf samples (0.5 g each) were divided into uniformly sized discs, rinsed three times in distilled water to get rid of surface-adhered electrolytes, and then put in two sets of 20 ml of distilled water. Using a conductivity meter (model CD-4301), one set was maintained at 40 °C for thirty minutes, and its electric conductivity in milli-Siemens (mS) was measured (EC1). The conductivity of the second set was likewise measured after it was placed in a bath of boiling water (100 °C) for 15 min (EC2). Thus, the formula for calculating MSI was as follows: MSI = (EC1/EC2) × 100.

### Membrane leakage (ML)

Vahala et al.^23^ method was applied to assess ML. Leaf samples (0.5 g each) were cut into uniformly sized discs, centrifuged for 80 min at 300 rpm, and then cleaned three times with distilled water to remove surface-adhered electrolytes. The samples were then put in 20 ml of pure water. The total ion leakage is expressed as a percentage of electric conductivity divided by fresh weight.

### Relative water content (RWC)

The Schonfeld et al.^24^ technique was used to measure RWC. The leaf’s center was pierced to release leaf discs. They were weighed in order to determine their fresh mass (FM), floated on distilled water for four hours, and then had another weigh-in to determine their turgid mass (TM). To determine the dry mass (DM), the discs were dried in an oven at 80 °C until their weight remained constant. The formula for calculating relative water content was RWC = (FM − DM)/(TM − DM) × 100.

### Saturation water deficit (SWD)

Schonfeld et al.^24^ method was used for measuring SWD based on the equation SWD % = 100 − RWC%

### Water use efficiency to yield (WUEG)

Hussain and Al-Jaloud^25^ method was utilized for calculating WUEG. The calculation of water use efficiency (kg/m^3^) involved dividing grain yield (kg/m^2^) by the total amount of water use for irrigation (m).

### Chlorophylls and carotenoids

Jichtenthaler and Buschmann^26^ method was used for calculating chlorophylls (chl a and chl b) and carotenoids. In an ice-cold porcelain mortar, a known fresh mass of flag leaves was chopped. To lessen acidity, several milligrams of Na_2_CO_3_ and some quartz sand were added. To compress the leaves, 1 ml of 80% acetone was powdered. Following the grinding process, the extract was mixed in a mortar with 3 ml of 80% acetone, and the pigment solution was then transferred into a centrifuge tube. In order to prevent acetone evaporation during centrifugation, the remaining pigment from the mortar was thoroughly mixed, rinsed multiple times with cold 80% acetone, and then the volume was reduced to 8 ml. The tube was then covered with parafin. The extract was centrifuged at 1000 xg for three minutes. Following centrifugation, the extracts were either sealed or kept in a cold, dark area, or the color was assessed right away. Using a spectrophotometer, the extract was measured (OD) at three different wavelengths: 480, 644, and 663 nm, in comparison to a blank of pure 80% aqueous acetone. Utilizing the following formulas, ascertain the pigment fraction concentrations (chl a, chl b, and carotenoids) as mg/g fresh weight of the plants that were subjected to different treatments: Chlorophyll a = (10.3 A663 − 0.918 A644), Chlorophyll b = (19.7 A644 − 3.87 A663), and Carotenoids = (5.02 A480).

### Nutrients content

The plant shoot with spike samples taken at heading, plant shoot samples without grains and grain samples taken at harvest were dried in an oven at 75 °C and the total K, total N and total P content were estimated according to^27^ . The zinc concentration in wheat grains was also determined using a flame atomic absorption spectrophotometer.

### Wet and dry gluten

Pleshkof^28^ method was used for measuring wet and dry gluten. In order to identify wet and dry gluten in fine air-dried grain, the meal was hand-washed until there was no more starch visible in the washing water, after which it was dried and weighed. Wet and dry gluten were estimated as percentage of air dry grains.

### Yield analyses

The heading date was calculated based on field observation, with the number of days from planting to flowering at fifty percent for the studied units.

The maturity date was measured from the date of planting to the plant’s physiological maturity.

Plant height (cm) the distance was measured from above ground to spike top at maturity.

Spike length (cm) the distance was calculated from the bottom of the spike to its highest point at maturity.

Spikes number/m^2^ were calculated after reaching full maturity for plants from the middle lines on the square meter.

The spike weight was weighed using the sensitive scale.

Grain number/spike were calculated using the mean grains number for ten spikes after manually severing these ears.

Hundred kernel weights (g) were counted randomly and weighed using the sensitive scale.

Biological yield (ts/fed) was estimated from the ten middle lines, i.e., distance within rows was 20 cm (2 × 3 m) after manual threshing of the plants from each experimental unit and the biological yield was extracted as ts/fed.

Grain yield (ts/fed) was calculated after separating the straw from the grains. The grains were weighed and calculated as ts/fed.

Straw yield (ts/fed) was estimated as follows: Straw yield = biological yield minus grain yield and it was expressed as ts/fed.

Harvest index was estimated by using the following equation: Grain yield / Biological yield.

### Statistical analysis

The data was submitted to analysis of variance (ANOVA) using CoHort/CoStat software, Version 6.311, the data was submitted to analysis of variance (ANOVA), and various letters reveal substantial variations between treatments at *p* ≤ 0.05.

## Results and discussions

To better understand physiological wheat adaptations to water stress, shoot and flag leaf biomass, chlorophyll and carotenoids content, RWC, SWD, WUEG, MSI, ML, nutrients content, yield, yield components and the chemical composition of wheat grains were studied. As demonstrated in Table 2, the soil at the experimental site exhibits deficiencies in nutrients before both wheat planting seasons, posing a nutritional problem for wheat crop production and grain nutritional content. Moreover, Table 3 described the experimental site’s climate during both wheat growing seasons.

### Impacts of zinc treatment on wheat growth characteristics under water stress

**Fig. 1:**
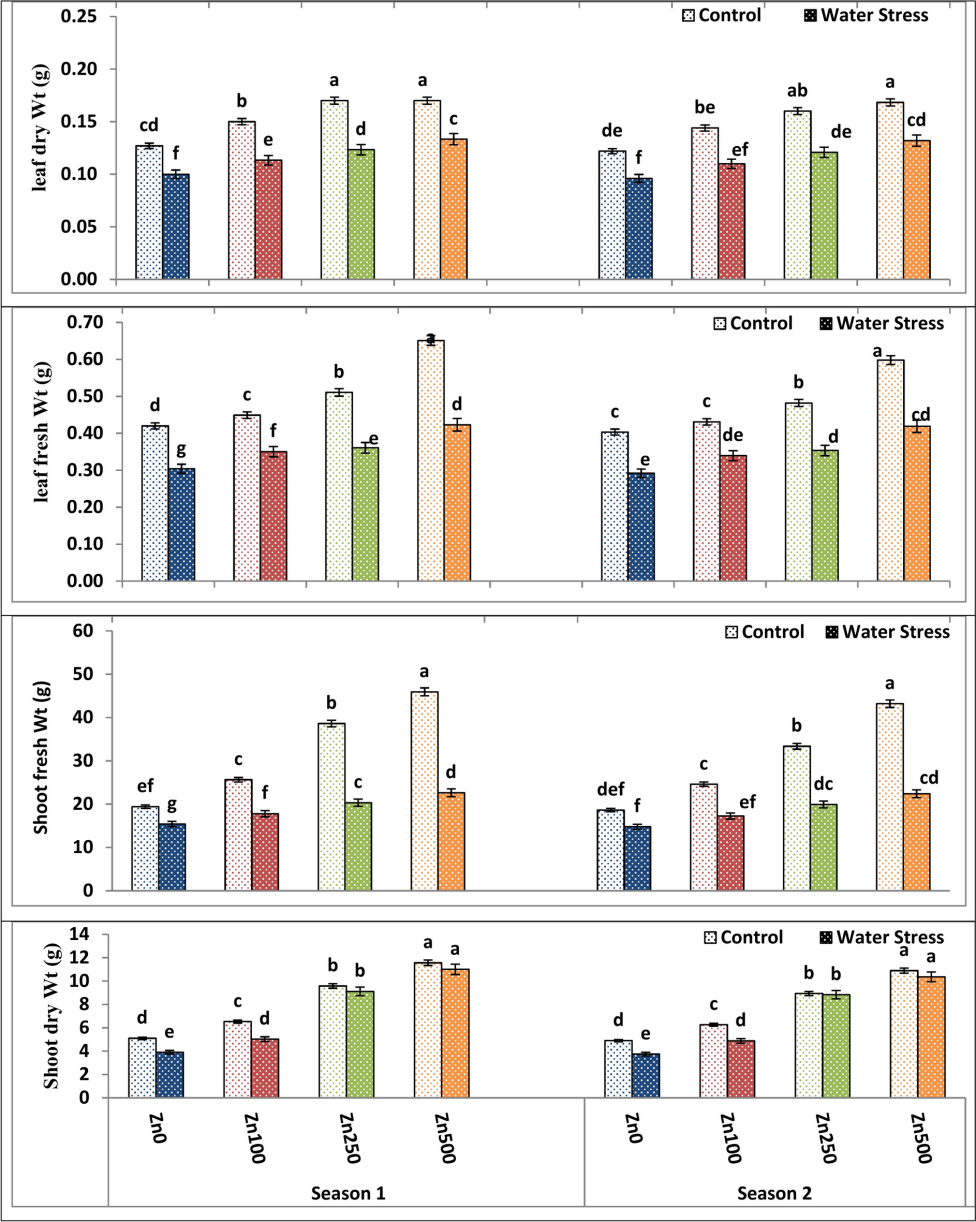
The impact of interactions between irrigation and Zn treatments on wheat shoot and flag leaf weights at heading. The vertical bars denote the standard error of the ensemble mean (n = 10). Different letters indicate significant differences between treatments at *p* ≤ 0.05.

**Fig. 2:**
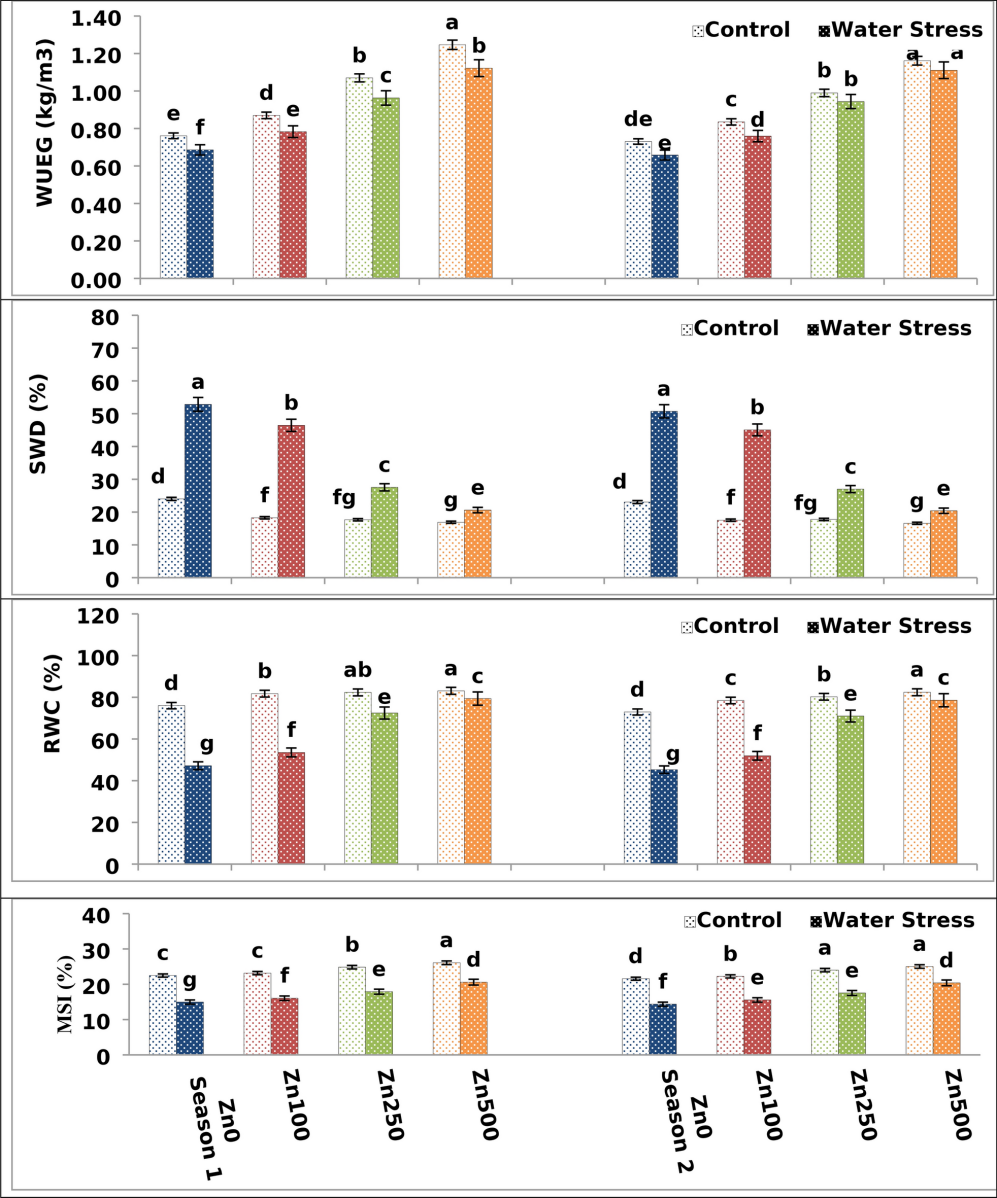
The impact of interactions between irrigation and Zn treatments on the membrane stability index (MSI), relative water content (RWC), saturation water deficit (SWD) and water use efficiency for grains (WUEG) of the wheat cultivar at heading. The vertical bars denote the standard error of the ensemble mean (n = 3). Different letters indicate significant differences between treatments at *p* ≤ 0.05.

As illustrated in Table 4 and Fig. 1, water treatment significantly affected shoot and flag leaf fresh and dry weight in both seasons. Water stress significantly declined shoot and flag leaf weight to 25.09–19.8% and 27.62–21.26% in both the first and second growing seasons, respectively. These results corroborated the Ghanem et al.^29^ findings who stated that drought decreased shoot and flag leaf growth in wheat plants. The highest values of the above-mentioned traits were produced by well-watering treatments, while the lowest means of them were given by the water stress treatment, this can be linked to a drop in tissue water content, turgor pressure, and cell turgidity as well as a fall in relative chlorophyll content, which stops cells from dividing and growing^30^. The results of this study lend credence to the hypothesis of Ghanem et al.^31^, which suggests that a decline in growth could be an adaptive trait that aids plants in surviving drought by storing the nutrients and energy required for shoot and leaf growth into molecules that function as barriers to the drought. Under water stress, plants could not uptake their water and nutrient requirements for plant development and various metabolism processes resulted in growth, cell division and organ progress declining. Ahmad et al.^32^ reported similar results. In this regard, Hatzig et al.^33^ stated that biomass reduction under stress conditions may indicate osmotic stress, which characterizes the first phase of stress. As a result, the decrease in shoot and flag leaf biomass observed under water stress could be attributed to changes in plant-water relations (Table 5 and Fig. 2). These changes might diminish meristem activity along with cell elongation, preventing cell expansion immediately following cell turgor pressure loss.

**Table 4:** The impact of water treatment and zinc level as well as the interaction between them on wheat shoot and flag leaf weights at heading.

Parameter	Shoot fresh Wt (g)		Shoot dry Wt (g)		Leaf fresh Wt (g)		Leaf dry Wt (g)	
Season	1	2	1	2	1	2	1	2
A. Irrigation								
Control	32.40	29.95	8.19	7.59	0.508	0.479	0.154	0.149
Water Stress	19.04	18.60	7.26	7.11	0.359	0.351	0.118	0.115
F test	**	*	*	*	*	*	*	*
B. Zn levels ppm								
0	17.41d	16.72d	4.50d	4.32d	0.362d	0.348dd	0.114c	0.109c
100	21.72c	20.94c	5.78c	5.57c	0.400c	0.385c	0.132b	0.127b
250	29.48b	26.65b	9.34b	8.88b	0.436b	0.418b	0.147a	0.140a
500	34.28a	32.80a	11.28a	10.62aa	0.537a	0.508a	0.152a	0.150a
F test	**	**	**	**	**	**	**	**
C. Interactions								
A × B	**	**	*	*	**	**	*	*

Based on CoHort/CoStat software, Version 6.311, various letters point to substantial variance between treatments at *p* 0.05, *significant at 0.05 possibility levels and **significant at 0.05 possibility levels.

**Table 5:** The impact of water treatment and zinc level as well as the interaction between them on wheat membrane stability index (MSI), membrane leakage (ML), relative water content (RWC), saturation water deficit (SWD), and water use efficiency for grains (WUEG) at the heading.

Parameter	ML (%)		MSI (%)		RWC (%)		SWD (%)		WUEG (kg/m^3^)	
Season	1	2	1	2	1	2	1	2	1	2
A. Irrigation										
Control	3.74	3.73	24.14	23.21	80.79	78.52	19.21	18.73	0.987	0.929
Water Stress	4.28	4.16	17.37	16.96	63.14	61.70	36.86	35.80	0.888	0.868
F test	*	*	**	*	**	**	**	**	**	ns
B. Zn levels ppm										
0	4.97a	4.77a	18.72d	17.97d	61.58d	59.11d	38.42d	36.89d	0.723d	0.694d
100	4.52b	4.37a	19.60c	18.90c	67.64c	65.21c	32.36c	31.29c	0.827c	0.797c
250	3.67c	3.62b	21.36b	20.77b	77.39b	75.62b	22.61b	22.38b	1.02b	0.967b
500	2.89d	3.02c	23.34a	22.71a	81.24a	80.50a	18.76a	18.50a	1.18a	1.14a
F test	**	**	**	**	**	**	**	**	**	**
C. Interactions										
A × B	ns	ns	*	*	**	**	**	**	*	*

Based on CoHort/CoStat software, Version 6.311, various letters point to substantial variance between treatments at *p* 0.05, *significant at 0.05 possibility levels and **significant at 0.05 possibility levels.

With respect to the Zn application effect, various zinc levels had a significant positive impact on both shoot and flag leaf fresh and dry mass in both growing seasons. Both shoot and leaf weight significantly responded to the gradual increase of Zn up to a high level of 500 ppm at which the latter Zn level gave the highest possible values of shoot and flag leaf weight of 39.14–43.49% and 33.33–37.50% for both seasons, respectively. On the contrary, the zero Zn level recorded the lowest values of shoot and leaf weight of 15.13–16.33% and 13.33–14.51% in first and second season, respectively (Fig. 1). The current finding is in line with the findings of Rai-Kalal et al.^34^, who reported that the essential role of zinc as a plant growth nutrient necessary for the proliferation of roots, increasing the uptake of water and other nutrients from the soil and delivering them to plant aerial parts, thereby enhancing the vegetative growth of plants, can explain the biomass reduction in Zn treated plants under stress.

**Fig. 3:**
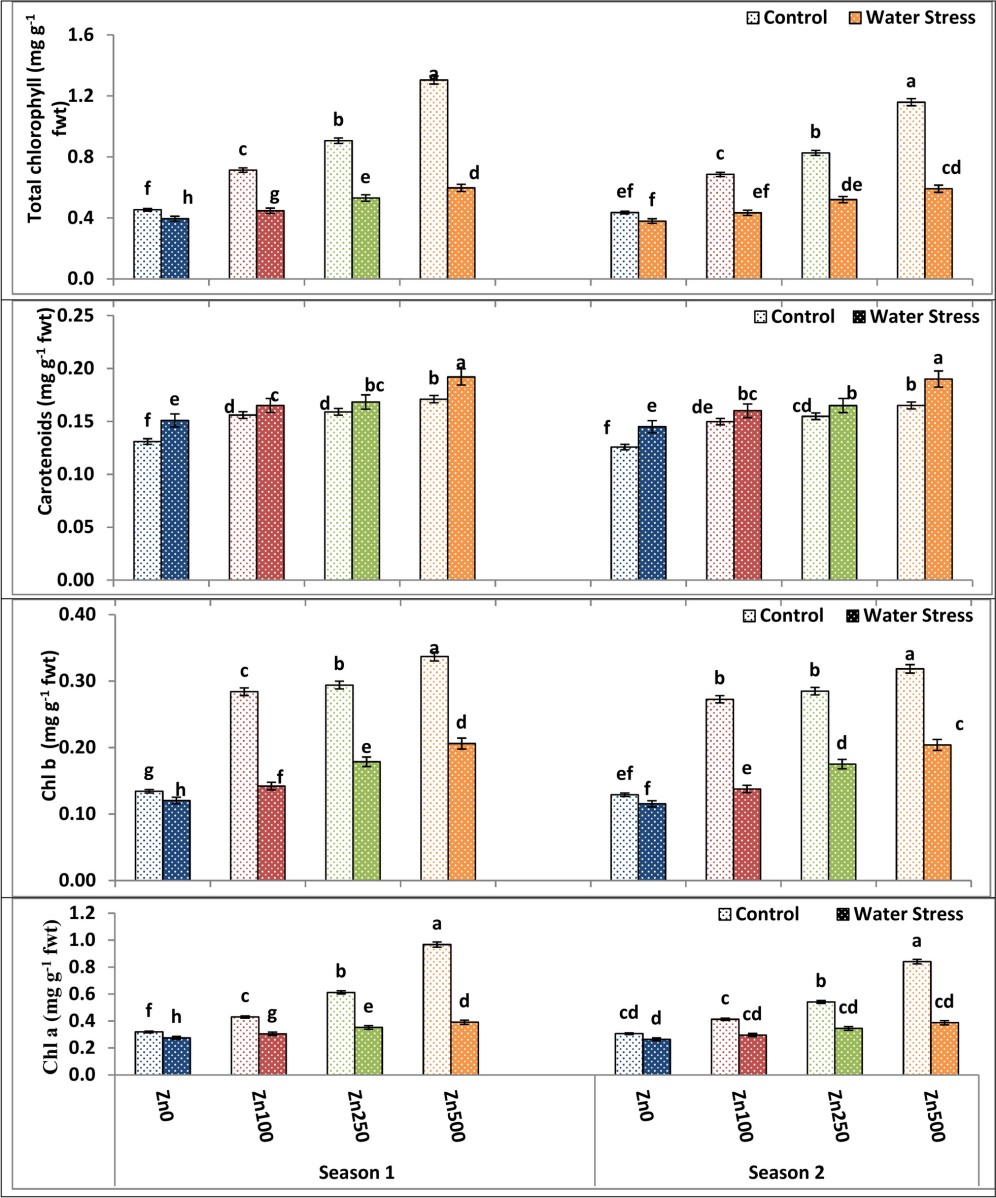
The impact of interactions between irrigation and Zn treatments on carotenoids, total chl, Chl a and Chl b of wheat cultivars at heading. The vertical bars denote the standard error of the ensemble mean (n = 3). Different letters indicate significant differences between treatments at *p* ≤ 0.05.

Our results are consistent with studies by Tolay^35^ and Hussein and Abou-Baker^36^, where zinc administration reduced the damage caused by water stress and enhanced plant development. According to Umair-Hassan et al.^37^, this suggests that zinc plays a critical role in activating a variety of enzymes linked to photosynthesis and metabolisms, which control cell division and growth. Furthermore, zinc functions as a co-enzyme with over 60 different enzymes. When higher dosages of zinc were administered during a drought, the plant’s resistance to the drought was increased, and growth characteristics and biomass were enhanced. The positive effect of Zn application was mainly attributed to the fact that Zn application results in an appreciable elevatation in carotenoids, total chl, chl, b and chl a contents as well as RWC (Figs. 2 and 3). Dimkpa et al.^38^ stated that Zn induced shoot and leaf growth by inducing cell elongation and growth. Moreover, several authors such as Dimkpa et al.^39^ came to similar results.

Regarding the interaction effect, the interaction between irrigation treatment and Zn application exerted a significant effect on shoot and leaf dry weight in both seasons. The best combination was a well-watered treatment with a high zinc level of 500 ppm since it gave the highest possible values of shoot and leaf weight (Fig. 1).

### Zinc application’s impact on physiological traits under water stress

#### Impacts of Zn treatment and water stress on membrane leakage (ML) and membrane stability (MSI)

The plant cell membrane is the primary focus of various abiotic stresses^40^ and maintaining its stability to withstand drought is a critical component of stress tolerance^41^. Table 5 and Fig. 2 showed that, when compared to control plants, water stress caused a further decrease in MSI by 31.12–33.42% and a significant increase in ML by 17.2–18.93% of wheat flag leaf at heading in both seasons, respectively. The highest values of MSI were produced by well watering treatment, while the lowest means of MSI were given by the water stress treatment. These findings are consistent with those of Ghanem et al.^29^ findings who stated that water stress induced a noticeable increase in ML and a clear decrement in MSI. Water stress causes peroxidation of membrane lipids, which causes essential solute leakage from the organelles and cells, causing impairments in numerous processes related to metabolism and membrane functions^42^.

With respect to the Zn application effect, various zinc levels had a significant positive effect on MSI and ML in both seasons of study. MSI significantly responded to the gradual increase of Zn up to the high level of 500 ppm, at which the latter Zn level gave the highest possible values of MSI by increasing 37.67–39.20% in both seasons. On the contrary, the zero Zn level recorded the lowest values of MSI (Fig. 2). These findings are consistent with those of Khan et al.^43^. In a drought, plants treated with trace metals such as zinc demonstrated improved water and turgor potentials^44^. In this respect, Sreenivasulu et al.^45^ proposed that adequate Zn supply maintains membrane permeability under water stress and preserves membrane lipids from ROS, thereby preventing ion channel leakage since Zn has unique properties because it exists in a divalent state lacking redox cycling and thus keeps stable in biological media and protects membrane lipids from ROS. According to Broadley et al.^46^, this improvement may be linked to reduced ion leakage and stable cell membranes, which enable plants to absorb more mineral nourishment when they are under water stress and ultimately improve the morphological characteristics of the plants.

In terms of interaction, the interaction between irrigation treatment and Zn application had a substantial impact on MSI across both seasons. The best combination was a well-watering treatment and a high zinc level of 500 ppm, which resulted in the highest MSI values (Fig. 1).

#### Responses of water relations to Zn application, and water stress

Plant water relations are directly affected by relative water content (RWC) and saturation water deficit (SWD). The RWC is a key physiological feature that determines a plant’s ability to withstand drought^47^. In the present investigation, water stress increased SWD by 45–47% while decreasing RWC by 36.2–37.93% and WUEG by 8.3–9.87% of wheat plants in first and second season, respectively (Table 5 and Fig. 2). This was consistent with research by Ghanem and Al-Farouk^8^, which showed that wheat plants exposed to drought had significantly lower RWC and WUEG values while having higher SWD values. Transpiration loss causes a reduction in the amount of water available to the soil, and when plants are stressed by drought, an unbalanced water status lowers plant RWC^48^.

In contrast, the foliar spraying of Zn at a rate of 500 ppm resulted in a significant increase in RWC by 68.32–73.58% and WUEG by 63.60–68.71% with a significant decrease in SWD by 60.99–59.77% when compared to control plants in both seasons, respectively. The higher Zn level exerted the highest values of RWC and WUEG. In terms of zero Zn foliar application, the control treatment had the lowest possible RWC and WUEG values. This result agreed with (Khan et al.^43^. In the other hand, plants’ RWC greatly increased when zinc fertilization rates were raised. Similar results were also observed in the rice crop^49^, where the use of exogenous zinc improved the relative water contents during water stress. It facilitates the plant’s increased root water absorption^50^. Hence, Zn foliar application enhances stomatal regulation by maintaining membrane integrity and improving drought tolerance by keeping leaf water content stable^51^. Furthermore, Zn application raises K^+^ influx in plant leaf guard cells, which improves stomatal conductance, resulting in noticeable WUE under water stress^37^. In general, the closing of stomata in response to drought enhances WUE and WUE because there is a greater reduction in transpiration than in the process of photosynthesis^52^.

Irrigation and Zn treatment had a cumulative interaction effect on RWC, WUEG and SWD as shown in Fig. 2. The combination between irrigation and Zn treatments of well watering and 500 ppm gave the highest possible values for RWC and WUEG and the lowest possible value of SWD (Fig. 3). On the other hand, the lowest possible values of RWC and WUEG and the highest values of SWD were produced by the combination of water stress treatment and zero Zn levels.

#### Impacts of Zn treatment, and water stress on photosynthetic pigments

The current results in Table 6 and Fig. 3 concisely demonstrated that water stress caused an enormous reduction in the chlorophyll contents (chlorophyll a by 14–15.22%, chlorophyll b by 10.42–11.55%), and the overall chlorophyll content by 12.94–13.15% of wheat flag leaf in both seasons . Meanwhile, drought enhanced the production of carotenoids to 16–15.27% in stressed wheat plants in both seasons. The well watering treatment produced the highest levels of chlorophyll and the lowest levels of carotenoids, whereas the water stress treatment of irrigation every four days produced the lowest levels of chlorophyll and the highest levels of carotenoids. stress significantly reduced the chlorophyll content of wheat flag leaf (total chlorophyll, Chl b and Chl a). At this point, water stress increased carotenoids production in stressed wheat plants. The outcomes were in line with the conclusions made by Ghanem et al.^29^ who demonstrated that drought caused a clear reduction in the chlorophyll contents along with an enormous increase in carotenoids content. Thus, the reduction can be attributed to ROS’s damaging effects on chloroplasts^53^. Mejri et al.^54^ stated that water stress alters the ratio of chlorophyll a to b and degrades it, hindering chloroplast activity. As a result, plants might be tempted to reduce their chlorophyll content in order to avoid the photooxidation process.

**Table 6:** The impact of water treatment and zinc level as well as the interaction between them on wheat flag leaf chlorophyll and carotenoids content at heading.

Parameter	Chl a (mg g^−1^ fwt)		Chl b (mg g^−1^ fwt)		Carotenoids (mg g^−1^ fwt)		Total Chl (mg g^−1^ fwt)	
Season	1	2	1	2	1	2	1	2
A. Irrigation								
Control	0.582	0.525	0.262	0.251	0.154	0.149	0.844	0.776
Water Stress	0.331	0.323	0.162	0.158	0.169	0.165	0.492	0.481
F test	**	*	**	*	**	*	*	*
B. Zn levels ppm								
0	0.297d	0.285c	0.127d	0.122d	0.141d	0.135d	0.424	0.407
100	0.367c	0.354c	0.213c	0.205c	0.161c	0.155c	0.580	0.559
250	0.482b	0.443b	0.236b	0.230b	0.164b	0.160b	0.718	0.673
500	0.679a	0.614a	0.272a	0.261a	0.182a	0.178a	0.951	0.875
F test	**	**	**	**	**	**	**	**
C. Interactions								
A × B	**	*	**	**	**	*	**	**

Based on CoHort/CoStat software, Version 6.311, various letters point to substantial variance between treatments at *p* 0.05, *significant at 0.05 possibility levels and **significant at 0.05 possibility levels.

The various Zn levels significantly affect wheat plant pigments in both seasons of study. Both chlorophyll and cartenoids significantly responded to the gradual increase of Zn up to the high level of 500 ppm at which the latter Zn level gave the highest possible values by increment of 42.53–46.9%, 71.19–76.54%, 27.15–31.13% and 51.27–55.99% for chl a, chl b, carotenoids, and total chl in both seasons, respectively. On the contrary, the zero Zn level recorded the lowest values of chlorophyll and carotenoids (Table 6 and Fig. 3). Furthermore, Zn application increases the content of chlorophyll and other photosynthetic pigments significantly^38^. Zn application enhances chlorophyll synthesis because Zn is the structural component of various proteins and enzymes as well as a co-factor for normal pigment biosynthesis^55^. Furthermore, Zn influences the concentration of nutrients involved in chlorophyll synthesis as well as the availability of other nutrients^56^. According to Arough et al.^57^, Zn can increase chlorophyll content and photosynthetic activity while enhancing plant growth.

In terms of interaction, the interaction among irrigation treatment and Zn application exerted a significant effect on total chl, chl b, chl a, and carotenoids content across both seasons (Table 4 and Fig. 3). The combination of well watering and Zn at the level of 500 ppm Zn application produced the highest values of total chl, chl b and chl a (Fig. 3). On the contrary, the combination of water stress treatment and a zero Zn level produced the lowest possible values of the aforementioned traits. Additionally, the highest value of carotenoids was produced under 500 ppm Zn application and water stress conditions (Fig. 3).

#### Impacts of Zn application, and water stress on nutrients content, and total protein

**Fig. 4:**
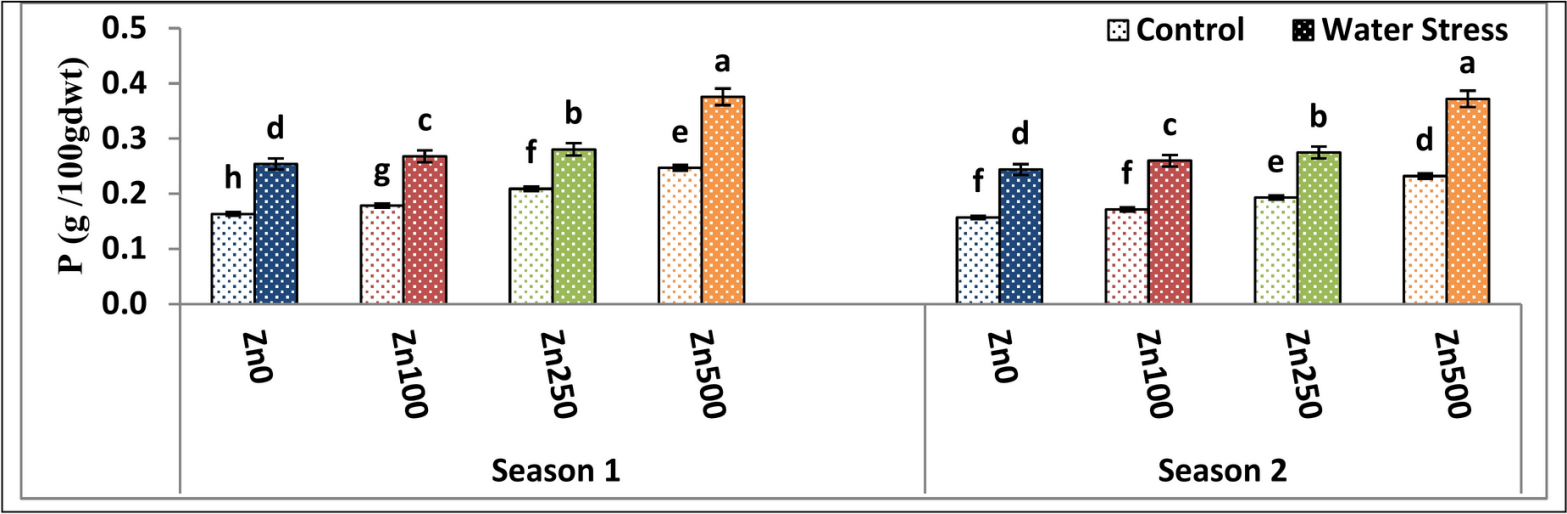
The impact of interactions between irrigation and Zn treatments on P content of wheat cultivar at heading. The vertical bars denote the standard error of the ensemble mean (n = 3). Different letters indicate significant differences between treatments at *p* ≤ 0.05.

As indicated in Table 7 and Fig. 4, water stress increased nitrogen, phosphorus, potassium and total protein content by 10.00,11.2%, 55.51–54.0%, 11.61–11.61% and 10.00–11.2 in wheat flag leaf at heading in both seasons, respectively. The stress treatment produced the highest values of the aforementioned traits, while the control treatment of irrigation every two days produced the lowest possible values. These findings run counter to those of previous studies conducted by Ghanem and Al-Farouk^8^. The accumulation of osmolytes in the form of N P K provides osmotic adaptation, which is the primary mechanism for plant tolerance to drought stress. In both normal and stressful environments, potassium (K), nitrogen (N), and phosphorus (P) function as osmoregulators and osmoprotectants in plants. So, RWC and MSI were enhanced by raising the K concentration in plants. Moreover, it’s possible that enhancing the synthesis of stress protein is what causes the rise in protein under water stress.

**Table 7:** The impact of water treatment and zinc level as well as the interaction between them on wheat flag leaf nitrogen, phosphorus, potassium content and total protein at heading.

Parameter	K (g/100gdwt)		N (g/100gdwt)		P (g/100gdwt)		Total protein (g/100gdwt)	
Season	1	2	1	2	1	2	1	2
A. Irrigation								
Control	2.27	2.18	0.252	0.242	0.200	0.188	1.58	1.53
Water Stress	2.54	2.44	0.321	0.308	0.295	0.288	2.01	1.96
F test	*	*	**	**	**	*	**	**
B. Zn levels ppm								
0	2.37c	2.28c	0.263c	0.252d	0.209d	0.200d	1.64c	1.58d
100	2.39b	2.30b	0.264c	0.254c	0.223c	0.216c	1.65c	1.59c
250	2.42a	2.33a	0.301b	0.289b	0.245b	0.234b	1.88b	1.84b
500	2.44a	2.35a	0.320a	0.307a	0.311a	0.302a	2.00a	1.97a
F test	**	**	**	**	**	**	**	**
C. Interactions								
A × B	ns	ns	ns	ns	**	**	ns	ns

Based on CoHort/CoStat software, Version 6.311, various letters point to substantial variance between treatments at *p* 0.05, *significant at 0.05 possibility levels and **significant at 0.05 possibility levels.

Furthermore, Zn application up to a rate of 500 ppm recorded the highest potassium, nitrogen, phosphorus, and total protein content in wheat flag leaf at heading by 3.08–2.98%, 40.12–44.50%, 47.90–52.52% and 40.12–44.50% in both seasons. Other nutrients, such NPK, were enhanced by Zn application^58^. Enhanced usage of applied N K P in the condition of Zn, which stimulated enhanced dry matter production and root growth, therefore leading to an improvement in phosphorus content, may be the cause of the rise in N K P content under water stress and Zn application. Moreover, it’s possible that enhancing the synthesis of stress protein is what causes the rise in protein under water stress and Zn application. Additionally, data on the total nitrogen contents in leaves revealed that Zn treatments increase the total nitrogen content and are connected with the synthesis of proteins, amino acids, and other macromolecules as well as the efficiency of nitrogen usage^59^. The accumulation of these osmolytes lowers cell water potential and increases water uptake under water stress^60^ and also safeguards sensitive cell components from oxidative damage^61^.

The same patterns were seen in our data on NPK contents, which we discovered to be higher in the Zn therapy cases. Zinc also helps stressed plants’ cell structure and morphological characteristics by regulating cell homeostasis^62^. Sun et al.^63^ observed that, in comparison to non-Zn treated plants, the concentration of soluble protein was higher in plants treated with zinc under both drought stress and non-stress circumstances. Reduced ion leakage, which lessens the damage brought on by drought stress, may be the reason of the increase in protein content following Zn application^64^.

In terms of interaction, the interaction among irrigation treatment and Zn application exerted a significant effect on P content and a non-significant effect on K and N content in flag leaf at heading across both seasons. The best combination was stress treatment and a high zinc level of 500 ppm since it gave the highest possible values of P content (Fig. 4).

### Plant phenology’s behavior to water stress and zinc treatment

Presumably, the current data (Table 8) clearly demonstrated that water stress caused a significant decrease in heading and maturity days. Hence, this reduction in heading and maturity days could be attributed to the reduction in the life span of plant leaves and, as a result, accelerated senescence, causing plants to mature early. These findings were consistent with those obtained by Bayoumi et al.^65^, who hypothesized that drought accelerates the growth phase and leads to a substantial decrease in the total number of days to heading.

**Table 8:** The impact of water treatment and zinc level as well as the interaction between them on heading, maturity, plant height, spikes number/m^2^, spike length and spike weight of wheat cultivar.

Parameter	Heading (days)		Maturity (days)		Plant height (cm)		No. of spikes/m^2^		Spike length (cm)		Spike weight (g)	
Season	1	2	1	2	1	2	1	2	1	2	1	2
A. Irrigation												
Control	70.25	68.17	123.92	120.36	99.0	95.74	299.75	287.76	13.28	12.75	3.92	3.73
Water stress	68.50	66.80	121.50	118.47	85.17	83.11	266.92	256.24	10.63	10.20	3.66	3.58
F test	*	*	*	*	*	*	*	*	**	**	*	*
B. Zn levels ppm												
0	67.83c	65.12d	121.33c	116.48d	84.83d	81.44c	245.00d	235.20d	7.98c	7.66d	2.98c	2.86c
100	68.83b	66.42c	122.17b	117.88c	91.67c	88.43b	265.50c	254.88c	12.21b	11.72c	3.74b	3.61b
250	70.00a	68.44b	123.50a	120.87b	93.83b	91.63b	295.00b	283.20b	13.45ab	12.91b	3.88b	3.80b
500	70.83a	69.96a	123.83a	122.43a	98a	96.20a	327.83a	314.72a	14.18a	13.62a	4.56a	4.35a
F test	**	**	**	**	**	**	**	**	**	**	**	**
C. Interactions												
A × B	ns	ns	ns	ns	*	*	**	**	*	*	*	*

Based on CoHort/CoStat software, Version 6.311, various letters point to substantial variance between treatments at *p* 0.05, *significant at 0.05 possibility levels and **significant at 0.05 possibility levels.

However, various levels of Zn significantly improved days to heading and maturity. The level of 500 ppm seemed to be the most successful in overcoming the adverse impacts of drought on heading and maturity days. Foliar application of Zn increased heading and maturity days since Zn enhanced total chlorophyll, Chl b, Chl a and carotenoids production in the leaf (Table 6 and Fig. 3) and, as a consequence, delayed senescence. Furthermore, irrigation and Zn treatment had no interaction effect on heading (days) and maturity (days) (Table 8).

### Yield and yield component responses to zinc treatments, and water stress

**Fig. 5:**
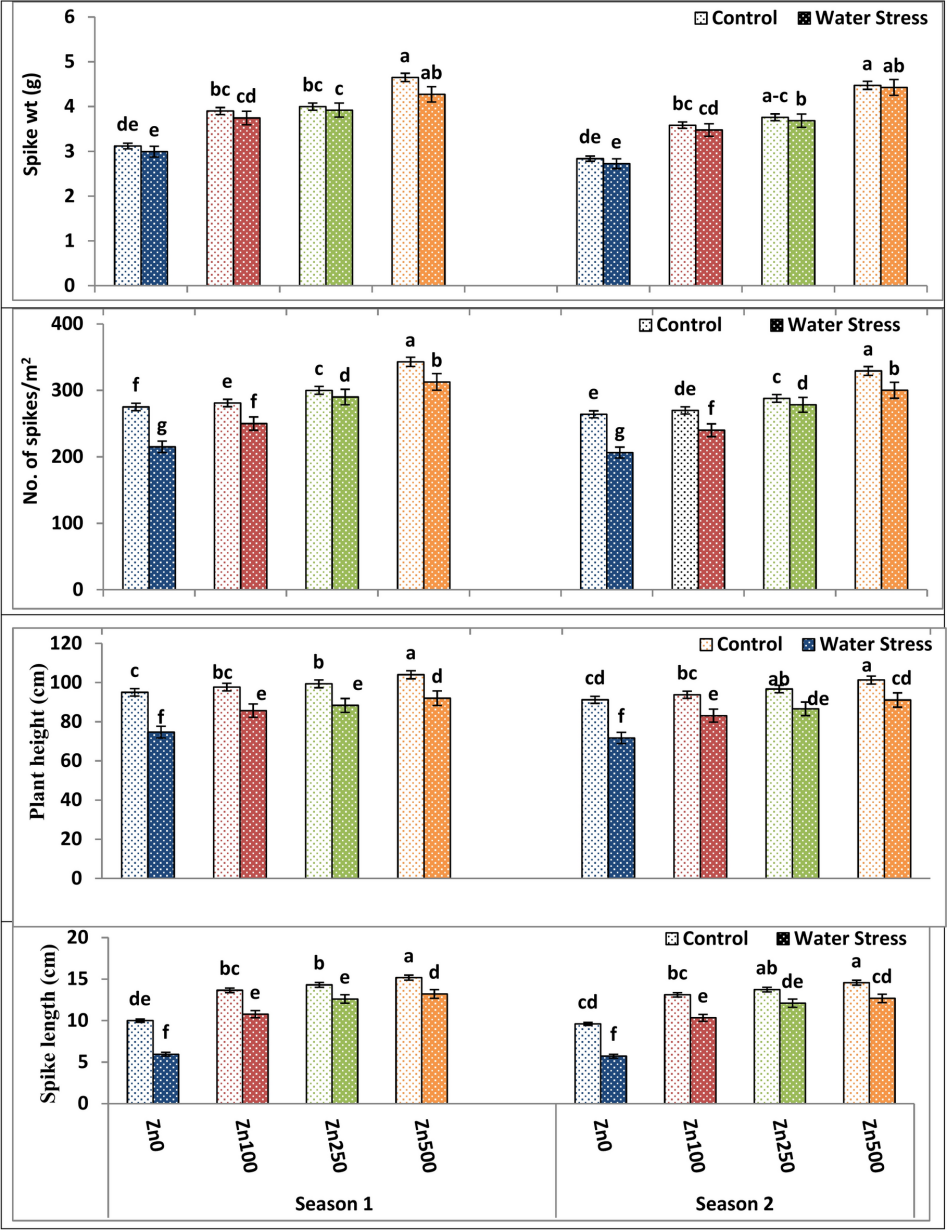
The impact of interactions between irrigation and Zn treatments on plant height, spikes number/m^2^, spike length and spike weight of wheat cultivar. The vertical bars denote the standard error of the ensemble mean (n = 3). Different letters indicate significant differences between treatments at *p* ≤ 0.05.

**Fig. 6:**
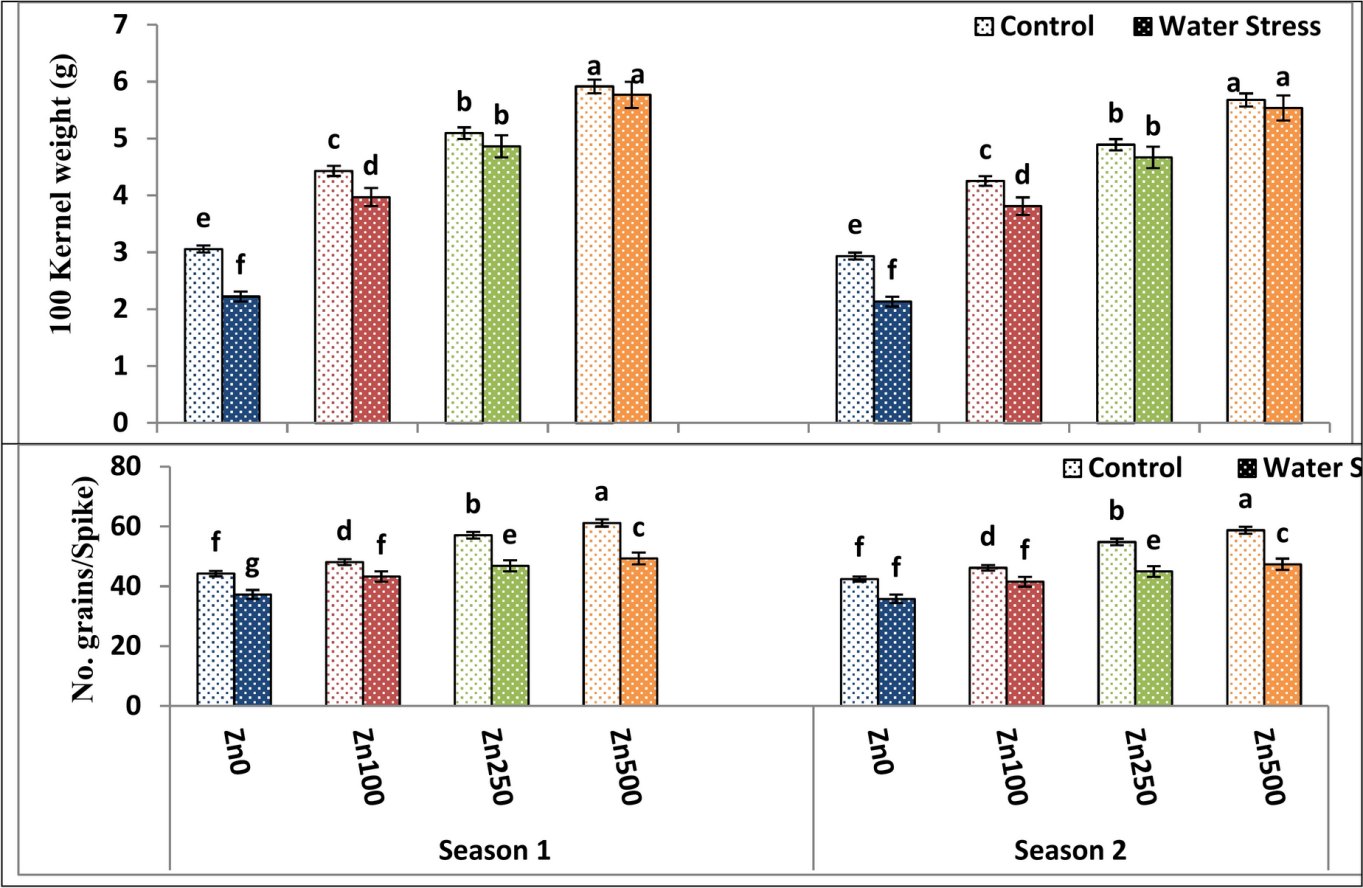
The impact of interactions between irrigation and Zn treatments on grain numbers/spike, and 100 kernel weight of wheat. The vertical bars denote the standard error of the ensemble mean (n = 3). Different letters indicate significant differences between treatments at *p* ≤ 0.05.

**Fig. 7:**
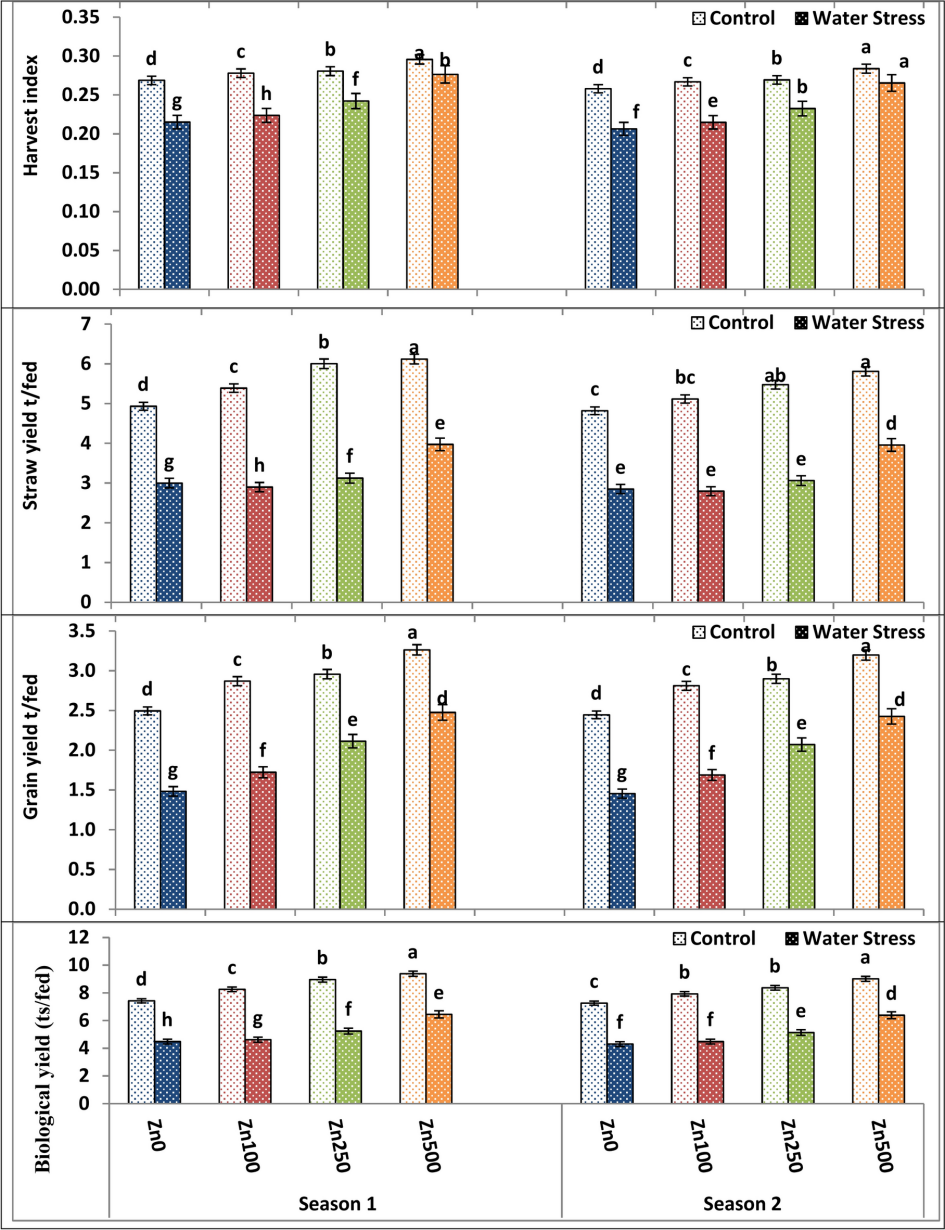
The impact of interactions between irrigation and Zn treatments on crop yield, grain yield, straw yield and harvest index of wheat. The vertical bars denote the standard error of the ensemble mean (n = 3). Different letters indicate significant differences between treatments at *p* ≤ 0.05.

As proved by Tables 8 and 9 and Figs. 5, 6 and 7, water treatment significantly affected yield and yield attributes in both seasons. Water stress significantly declined plant height (21.82–23.1%), spike length (40.50–41.3%), main spike weight (9.08–10.12%), grain number/spike (15.7–14.32%), 100 kernel weight (27.30–25.6%), biological yield (39.66–40.77%), straw yield (40.57–39.20%) as well as grain yield (40.57–49.86%) and harvest index (19.98–20.2%) of wheat plants among both the first and second seasons, respectively. Drought significantly reduced the grain yield of untreated plants in the current study, owing to the lower grain weight and number of grains. The highest values of the above-mentioned traits were produced by the well watering treatment, while the lowest means of them were given by the stress treatment. These findings are consistent with those of Anwar et al.^66^, who found that diminished grain yield in wheat plants under water stress was primarily due to a decrease in the number of grains and grain weight.

**Table 9:** The impact of water treatment and zinc level as well as the interaction between them on grain number/spike, 100 kernel weight, biological yield, straw yield as well as grain yield and harvest index of wheat cultivar.

Parameter	No. grains/Spike		100 Kernel weight (g)		Biological yield ts/fed		Grain yield t/fed		Straw yield t/fed		Harvest index	
Season	1	2	1	2	1	2	1	2	1	2	1	2
A. Irrigation												
Control	52.62	50.51	4.62	4.44	8.51	8.14	2.90	2.84	5.61	5.31	0.281	0.269
Water Stress	44.15	42.39	4.21	4.04	5.20	5.07	1.95	1.91	3.25	3.17	0.239	0.230
F test	*	*	ns	ns	**	*	**	**	**	*	**	*
B. Zn levels ppm												
0	40.73d	39.10d	2.64d	2.53d	5.95d	5.78d	1.99d	1.95d	3.97d	3.83d	0.242d	0.232d
100	45.65c	43.82c	4.20c	4.03c	6.44c	6.20c	2.30c	2.25c	4.14c	3.95c	0.251c	0.241c
250	51.93b	49.86b	4.98b	4.78b	7.10b	6.75b	2.54b	2.48b	4.56b	4.27b	0.261b	0.251b
500	55.23a	53.02a	5.84a	5.61a	7.92a	7.70a	2.87a	2.81a	5.05a	4.88a	0.286a	0.275a
F test	**	**	**	**	**	**	**	**	**	**	**	**
C. Interactions												
A × B	**	**	*	*	**	*	**	**	**	*	**	**

Based on CoHort/CoStat software, Version 6.311, various letters point to substantial variance between treatments at *p* 0.05, *significant at 0.05 possibility levels and **significant at 0.05 possibility levels.

Grain yield is measured by its distinct characteristics, which include spike number /m^2^, grain number/spike, and 100 kernel weights. As a result, grain number/spike and grain weight are significant components of wheat grain yield that are influenced by drought. Water stress can reduce grain yield by as much as 50% through diminished grain numbers^67^. The decrease in shoot and flag leaf biomass (Table 4 and Fig. 1), plant water relations (Table 5 and Fig. 2), membrane characteristics (Table 5 and Fig. 2), and a photosynthetic pigment (Table 6 and Fig. 3) can all be attributed to the reduction in grain yield of stressed wheat plants.

In both seasons of study, there was substantial variation in wheat yield and yield attributes among zero Zn spray plants and sprayed plants under both control and water stress conditions. Data, as shown in Tables 8 and 9 and Figs. 5, 6 and 7, showed that foliar application of Zn had a significant and beneficial impact on wheat yield as well as yield attributes in both the first and second growing seasons, respectively. Spikes number/m^2^, spike length, plant height, main spike weight, grain number/spike, 100 kernel weight, biological yield, straw yield as well as grain yield and harvest index of wheat plants were all substantially raised through elevating Zn foliar spray from 100 to 500 ppm in both the first and second growing seasons, with the latter Zn level giving the best possible values of the above-mentioned traits. On the contrary, the zero Zn level had the lowest possible values of these traits (Tables 8 and 9 and Figs. 5, 6 and 7). The increased grain yield by 66.95–65.3% obtained in the current study as a result of Zn application coincides with increased 100 kernel weight by 78-0.71–75.3% and grain number/spike by 32.31–36.8% in first and second season, respectively. These findings were consistent with Sattar et al.^68^. Furthermore, Chattha et al.^69^ reported that Zn application increased maize yield and harvest index under water stress conditions. Previous investigations also demonstrated that Zn foliar application increased yield^70^. The general pattern of plant response to Zn foliar application and stress is yield improvement, where thus improvement could attributed to the fact that Zn foliar application enhanced shoot and flag leaf biomass (Table 4 and Fig. 1), plant water-relations (Table 5 and Fig. 2), membrane characteristics (Table 5 and Fig. 2), a photosynthetic pigment (Table 6 and Fig. 3).

In terms of interaction, the interaction between irrigation treatment and Zn application exerted a significant effect on shoot and leaf dry weight across both seasons. The best combination was a well-watered treatment with a high zinc level of 500 ppm since it gave the highest possible values of yield and yield components (Tables 8 and 9 and Figs. 5, 6 and 7).

### Effects of water stress and zinc on NPK of wheat grains and straw

**Fig. 8:**
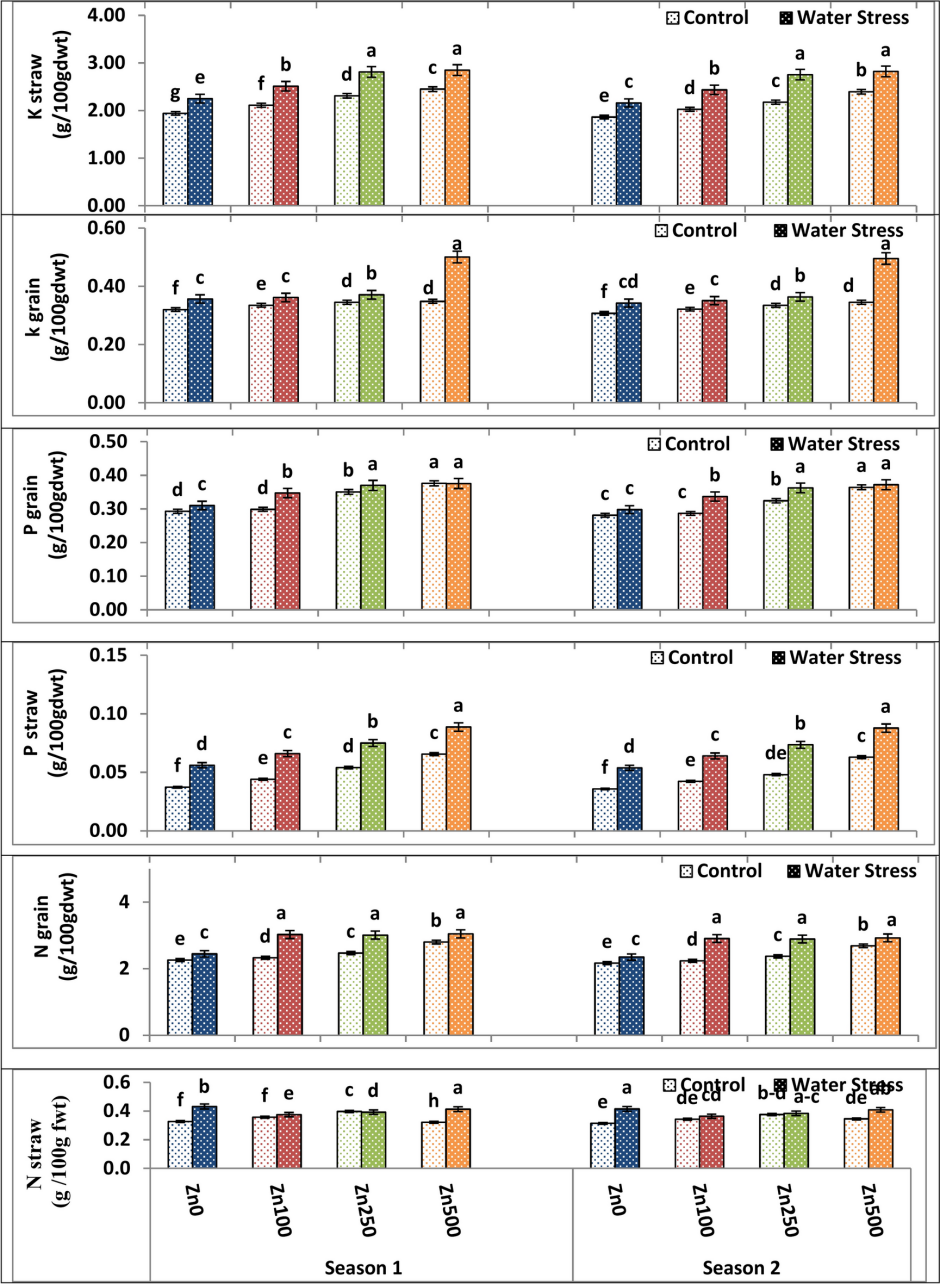
The impact of interactions between irrigation and Zn treatments on nitrogen, phosphorus and potassium content in grain and straw of wheat. The vertical bars denote the standard error of the ensemble mean (n = 3). Different letters indicate significant differences between treatments at *p* ≤ 0.05.

Water stress increased the concentration of NPK content in stressed wheat grains along with straw in both growing seasons, as shown in Table 10 and Fig. 8. The highest values of NPK content were produced by the stress treatment, while the lowest values were given by the control treatment. It’s possible that the observed rise in NPK content under water stress represents an adaptation mechanism meant to boost cellular osmotic pressure and aid in maintaining more water. With respect to Zn application effect, various zinc levels had a significant positive effect on NPK content in both seasons of study. Both of straw and grains of wheat significantly responded to gradual increase of Zn up to the high level of 500 ppm in which the latter Zn level gave the highest possible values of NPK content. On the contrary, the zero Zn level recorded the lowest possible values of NPK content (Fig. 8). Therefore, the increase in NKP in grain and straw under water stress and Zn treatment may be the result of better fertilizer usage. Also, the increased grain NPK levels caused by Zn foliar application confirm its role in enhancing plant tolerance by increasing osmolyte accumulation. Conversely, as belowground roots primarily take up water and nutrients from the soil, the rise in grain and straw NKP under water stress with and without Zn application may be to meet plant needs and boost plant growth, which considerably enhances crop yields^71^. In terms of interaction, the interaction between irrigation treatment and Zn application exerted a significant effect on NPK content in the grains and the straw of the wheat plants across both seasons. The best combination was stress treatment and a high zinc level of 500 ppm since it gave the highest possible values of NPK content in grains and straw of wheat plants (Fig. 8).

**Table 10:** The impact of water treatment and zinc level as well as the interaction between them on nitrogen, phosphorus and potassium content in grain and straw of wheat cultivar.

Parameter	k grain (g/100gdwt)		K straw (g/100gdwt)		N grain (g/100gdwt)		N straw (g/100gdwt)		P grain (g/100gdwt)		P straw (g/100gdwt)	
Season	1	2	1	2	1	2	1	2	1	2	1	2
A. Irrigation												
Control	0.337	0.327	2.20	2.11	2.47	2.37	0.351	0.345	0.330	0.314	0.050	0.047
Water Stress	0.398	0.388	2.61	2.54	2.88	2.77	0.403	0.393	0.351	0.342	0.071	0.070
F test	*	**	**	*	**	**	**	*	*	*	*	*
B. Zn levels ppm												
0	0.338d	0.325d	2.10d	2.01d	2.35d	2.26d	0.380c	0.353d	0.302d	0.290d	0.047d	0.045d
100	0.348c	0.336c	2.31c	2.23c	2.68c	2.57c	0.366c	0.364c	0.323c	0.312c	0.055c	0.053c
250	0.358b	0.349b	2.56b	2.46b	2.74b	2.63b	0.395b	0.377b	0.360bb	0.343b	0.065b	0.061b
500	0.425a	0.420aa	2.65a	2.61a	2.92a	2.81a	0.368a	0.380a	0.376a	0.368a	0.077a	0.075a
F test	**	**	**	**	**	**	**	**	**	**	**	**
C. Interactions												
A × B	**	**	*	*	**	**	**	*	**	*	*	*

Based on CoHort/CoStat software, Version 6.311, various letters point to substantial variance between treatments at *p* 0.05, *significant at 0.05 possibility levels and **significant at 0.05 possibility levels.

### Effects of water stress and zinc on Zn, total protein, wet and dry gluten content of wheat grains

**Fig. 9:**
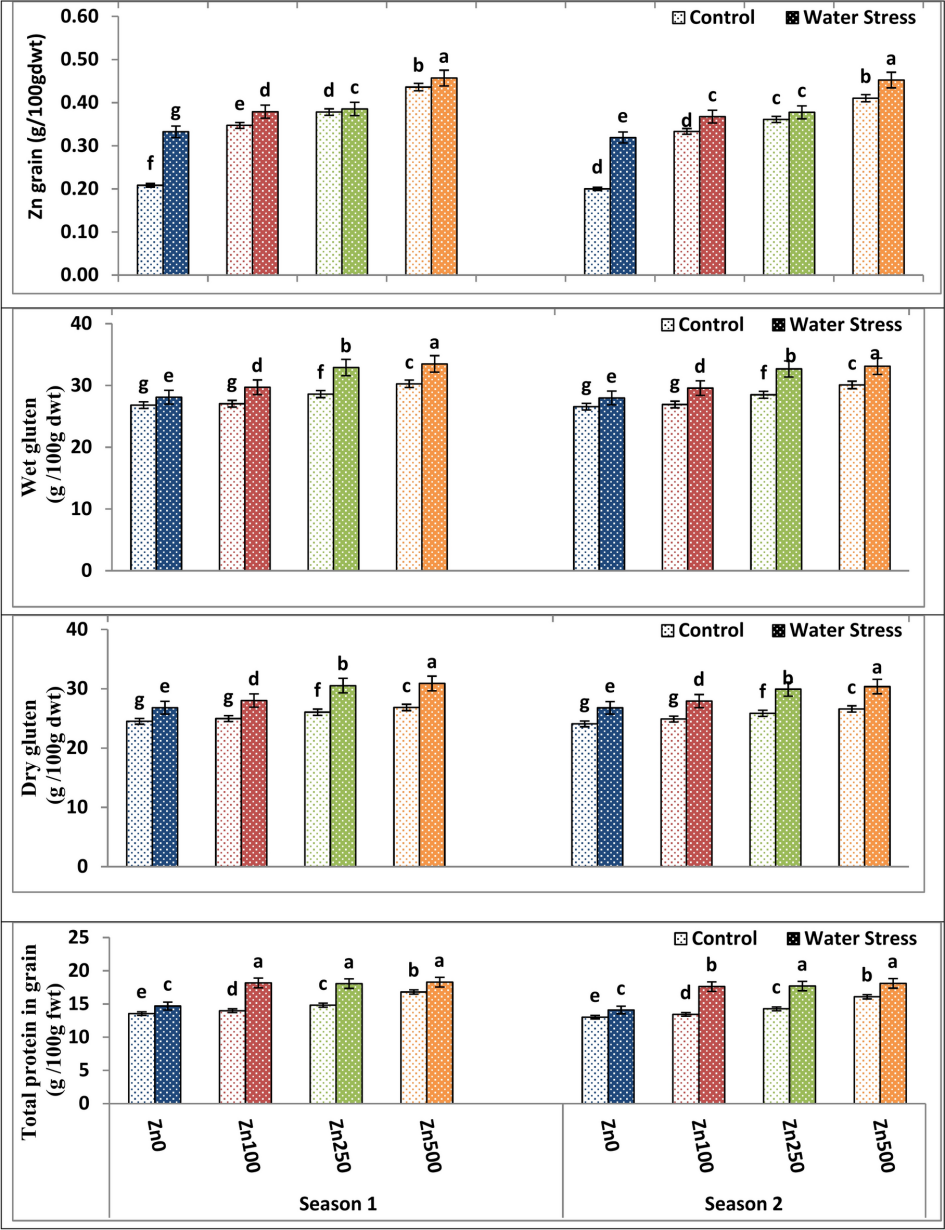
The impact of interactions between irrigation and Zn treatments on Zn, total protein, wet and dry gluten content in wheat grains. The vertical bars denote the standard error of the ensemble mean (n = 3). Different letters indicate significant differences between treatments at *p* ≤ 0.05.

According to the current findings, as shown in Table 11 and Fig. 9, water stress caused a significant increase in Zn, total protein, wet and dry gluten content by 37.34–35.2%, 8.26–9.12%, 17.86–21.52%, and 50.69–66.38% in wheat grain in both seasons, respectively. The stress treatment produced the best values of the aforementioned traits, while the control treatment produced the worst. Also, results clearly indicated that drought had a positive effect on wet and dry gluten. Thus, the results clearly indicate that drought induced the formation of greater amounts of gluten. This raised gluten content could be due to those with appropriate phenotypic features^72^.

**Table 11:** The impact of water treatment and zinc level as well as the interaction between them on Zn, total protein, wet and dry gluten content in wheat grains.

Parameter	Zn grain (g/100 gdwt)		Total protein (g/100 gdwt)		Wet gluten (g/100 gdwt)		Dry gluten (g/100 gdwt)	
Season	1	2	1	2	1	2	1	2
A. Irrigation								
Control	0.357	0.349	14.79	14.20	28.21	28.01	25.60	25.36
Water stress	0.374	0.356	17.30	16.88	31.09	30.83	29.06	28.75
F test	**	**	**	*	**	**	**	**
B. Zn levels ppm								
0	0.270d	0.259d	14.12d	13.56d	27.48d	27.27d	25.67d	25.43d
100	0.363c	0.350c	16.07c	15.52c	28.43c	28.24c	26.49c	26.41c
250	0.382b	0.369b	16.44b	15.98b	30.80b	30.58b	28.29b	27.89b
500	0.447a	0.431a	17.54a	17.09a	31.88a	31.60a	28.88a	28.48a
F test	**	**	**	**	**	**	**	**
C. Interactions								
A × B	**	**	**	**	**	**	**	**

Based on CoHort/CoStat software, Version 6.311, various letters point to substantial variance between treatments at *p* 0.05, *significant at 0.05 possibility levels and **significant at 0.05 possibility levels.

Furthermore, different levels of Zn significantly increased Zn, total protein, wet and dry gluten content by 82.01–83.90%, 24.54–28.43%, 66.71–64.55%, 59.99–52.81% in wheat grains in both seasons, respectively. In general, Zn application, specifically at 500 ppm, seemed to be the best option for overcoming the adverse impacts of drought on the above-mentioned parameters (Table 11 and Fig. 9). As a result, an increase in grain total protein content could be attributed to the synthesis of stress proteins, which provide the adaptation that constitutes the primary mechanism for crops tolerance to water stress. Because most stress proteins are water-soluble, they contribute significantly to stress tolerance mechanisms via hydrating cell structures^73^. Also, enhanced grain Zn via Zn foliar application affirms its role in human nutrition and food fortification. These findings are consistent with those of Hong and Ji-Yun^74^ and^75^. As demonstrated by^76^, improved Zn uptake with foliar Zn application may be due to the high Zn content and enhanced bioavailability of Zn within soil as well as plant systems.

In terms of interaction, the interaction between irrigation treatment and Zn application exerted a significant effect on dry and wet gluten as well as total protein and Zn contents across both seasons. The best combination was stress treatment and a high zinc level of 500 ppm since it gave the highest possible values of Zn, total protein, wet and dry gluten content (Fig. 9).

## Conclusion

The current findings show that Zn-treated wheat plants successfully enhanced tolerance under drought. As a result, we advocate spraying bread wheat with Zn (500 ppm) during the growing season for better wheat grain quality and production by enhancing the relative content of wet and dry gluten, total protein, and NPKZn in wheat grains. Indeed, grain accumulation of NPKZn nutrients was critical for enhancing the nutritional value of grains for human health. The current research offers valuable insights into the physiological and biochemical processes that underlie zinc foliar application and plant stress tolerance. Additionally, this study shows that using zinc under water stress can improve grain yield and quality. Based on the current study and according to Egypt’s agro-ecological circumstances, farmers should be encouraged to apply Zn as a foliar spray for wheat on drought-prone soils.

## Data Availability

The authors confirm that the data supporting the findings of this study are available within the manuscript.

## Declarations

### Plant guideline

The source of the wheat grains was the Wheat Research Department, Field Crops Research Institute, Agricultural Research Center, Egypt. The plant material comply with relevant institutional, national, and international guidelines and legislation.
